# Nullspace Sampling with Holonomic Constraints Reveals Molecular Mechanisms of Protein Gαs

**DOI:** 10.1371/journal.pcbi.1004361

**Published:** 2015-07-28

**Authors:** Dimitar V. Pachov, Henry van den Bedem

**Affiliations:** 1 Department of Chemistry, Stanford University, Stanford, California, United States of America; 2 Joint Center for Structural Genomics, Stanford Synchrotron Radiation Lightsource, Stanford University, Stanford, California, United States of America; University Of Oxford, UNITED KINGDOM

## Abstract

Proteins perform their function or interact with partners by exchanging between conformational substates on a wide range of spatiotemporal scales. Structurally characterizing these exchanges is challenging, both experimentally and computationally. Large, diffusional motions are often on timescales that are difficult to access with molecular dynamics simulations, especially for large proteins and their complexes. The low frequency modes of normal mode analysis (NMA) report on molecular fluctuations associated with biological activity. However, NMA is limited to a second order expansion about a minimum of the potential energy function, which limits opportunities to observe diffusional motions. By contrast, kino-geometric conformational sampling (KGS) permits large perturbations while maintaining the exact geometry of explicit conformational constraints, such as hydrogen bonds. Here, we extend KGS and show that a conformational ensemble of the α subunit Gαs of heterotrimeric stimulatory protein Gs exhibits structural features implicated in its activation pathway. Activation of protein Gs by G protein-coupled receptors (GPCRs) is associated with GDP release and large conformational changes of its α-helical domain. Our method reveals a coupled α-helical domain opening motion while, simultaneously, Gαs helix α_5_ samples an activated conformation. These motions are moderated in the activated state. The motion centers on a dynamic hub near the nucleotide-binding site of Gαs, and radiates to helix α_4_. We find that comparative NMA-based ensembles underestimate the amplitudes of the motion. Additionally, the ensembles fall short in predicting the accepted direction of the full activation pathway. Taken together, our findings suggest that nullspace sampling with explicit, holonomic constraints yields ensembles that illuminate molecular mechanisms involved in GDP release and protein Gs activation, and further establish conformational coupling between key structural elements of Gαs.

## Author Summary

Multi-cellular physiology is an emergent property, which depends critically on inter-cellular signaling pathways. Transmembrane G protein-coupled receptors (GPCRs) mediate a large variety of physiological events throughout the body, such as vision or cardiovascular regulation. It is thus no surprise that GPCRs are targeted by more than one third of all FDA-approved drugs. Molecules such as hormones and neurotransmitters transmit messages to cells via GPCRs complexed to cytosolic heterotrimeric G proteins. G proteins, upon activation, interact with other molecules to trigger a cellular response. Despite an increasing amount of structural data, the precise conformational dynamics and activation mechanism of G proteins remain poorly understood. The size of the multi-protein complexes and the time scales at which conformational changes occur hinder adequate sampling of the conformational landscape with molecular dynamics simulations. Here, we extend and use an efficient, robotics-inspired conformational sampling procedure to probe the conformational landscape of protein G during activation. Our procedure reveals coupled, molecular mechanisms of the activation pathway, which are absent in a comparative analysis with normal modes. Our exciting results can ultimately lead to modulation of biological activity by drug design or fine-tuning of conformational heterogeneity.

## Introduction

G protein-coupled receptors (GPCRs) mediate a large variety of physiological events throughout the body by activating intracellular signal transduction pathways [1]. Signaling molecules, such as hormones and neurotransmitters, can induce conformational changes in GPCRs, which allow it to complex with intracellular protein partners such as heterotrimeric guanine nucleotide-binding protein G. *β*_2_ Adrenergic Receptor (*β*_2_AR), a so-called class A receptor, initiates activation of stimulatory protein Gs mainly through interactions with the latter’s *α* subunit (G*α*s). Upon activation, Gs interacts with effector proteins in the cell which, ultimately, leads to a cellular response. However, a precise characterization of the activation mechanism of Gs has remained elusive [2].

Molecular dynamics (MD) simulations can structurally characterize the dynamics of biomolecules in great detail [3]. However, as increasingly sophisticated experimental techniques yield ever bigger molecular systems and complexes, the computational demands to ensure adequate sampling of the conformational landscape often require highly specialized hardware and algorithms [4].

In parallel, time-independent or non-deterministic sampling-based algorithms together with simplified macromolecular representations have also led to tremendous insights. Conformational sampling with CONCOORD has provided seeds for subsequent MD simulations to overcome large energy barriers in the characterization of recognition dynamics of ubiquitin [5, 6]. Rapid exploration of conformational space in internal coordinates with a traditional mechanical force field via a biased Monte Carlo approach [7] accurately predicted agonist binding modes for GPCRs [8]. Exhaustive sampling has predicted ensembles of low-energy conformers for GPCRs associated with ligand binding and activation [9]. Rosetta-based sampling and energy analysis provided a structural basis for rhodopsin-mediated GDP release from Gi, a inhibitory protein highly related to Gs [10].

Vibrational modes of a biomolecule are well-approximated with a so-called Elastic Network Model (ENM), in which non-bonded interactions are replaced with a harmonic pseudo-potential [11]. Analysis of ENMs with NMA, which relies on a Hamiltonian in which the kinetic energy is also quadratic, yields the equations of motion around a minimum of the potential energy of the system. While low-frequency modes are generally associated with biological activity, the second order approximation underlying NMA limits its ability to access conformational substates and observe larger, diffusional motions. Nonetheless, NMAs are enormously successful and have, for instance, proposed GPCR activation mechanisms [12]. Combined with short MD trajectories NMA also predicted a molecular mechanism for GDP release from Gi [13].

Kinogeometric sampling (KGS) treats a biomolecule as a branched polymer, with rotatable bonds as degrees of freedom (DoFs) and non-covalent (hydrogen) bonds as distance constraints [14–16]. Hydrogen bonds define nested, closed loops that require coordinated changes of DoFs to avoid breaking the bonds. Kinogeometric sampling maps structural perturbations onto a subspace of conformation space that accounts for the reduced flexibility of these closed loops. This procedure intrinsically favors certain directions on the conformational landscape, namely those that avoid, collectively, native hydrogen bond dissociation. Additionally, representing biomolecular systems with fewer DoFs enables better exploration of conformation space and, ultimately, allows fitting sparse experimental data sets while reducing the risk of overfitting.

Distance constraints from hydrogen bonds can completely rigidify substructures of biomolecules. For instance, an *α*-helix is often rigidified owing to its backbone hydrogen bonding network. Kinogeometric and similar sampling-with-constraint techniques have relied on combinatorial constraint counting to explicitly identify rigid substructures in the molecule that result from the hydrogen bonds [17]. Perturbing a molecular conformation with constraints generally required breaking constraints and subsequently reclosing them [18], or iteratively refitting the perturbed conformation and the rigid substructures [19].

Here, we extend our kinogeometric computational techniques by abandoning explicit constraint counting to proteins. Our procedure efficiently samples conformational degrees of freedom in a lower-dimensional subspace in which instantaneous distance constraints are preserved exactly [20]. The advantage of our method is that a single, exact mathematical analysis both provides constraint satisfaction and infinitesimal, coordinated directions of motion for the degrees of freedom of the protein. It naturally couples motions throughout the protein by many interconnected and interdependent cycles, making few additional assumptions on interactions. As a result, collective motions emerge which deform the protein along preferred dimensions. We apply our algorithm to compute a broad conformational distribution of the inactive and active states of the *α* subunit of free (i.e. not receptor-bound), apo (i.e. nucleotide-free) G*α*s. We demonstrate that our ensemble identifies detailed molecular mechanisms implicated in domain opening and activation of protein Gs. We compare the findings to an ensemble obtained with a state-of-the art torsional ENM. An ENM representation with torsional degrees of freedom is conceptually similar to our approach, and is known to better represent protein conformational changes than Cartesian ENMs [21, 22]. We selected an implementation, the iMC module of iMod, that is capable of generating large domain motions by sampling along low-frequency normal modes [23].

## Methods

### A kinematic representation of proteins

The linear, branched structure of proteins naturally forms a kinematic linkage, i.e. a chain with rigid groups of atoms, or rigid bodies, as links and rotatable bonds or degrees of freedom (DoF), as revolute joints. The DoFs are the backbone torsion angles (*ϕ* and *ψ*) and the side-chain torsion angles (*χ*_*i*_). Bond lengths, bond angles and the peptide torsion angle *ω* are assumed fixed at their initial values in this study. Rigid bodies are the largest sets of atoms in a protein without internal, rotational degrees of freedom (S1 Fig). We initially set each atom or group of double-bonded atoms as a rigid body. The rigid bodies of atoms connected by a non-rotatable covalent bond are merged. Hydrogen atoms are explicitly included in the model. A vector **q** ∈ 𝕊^*n*^,**q** = (*q*_1_, …, *q*_*n*_)^*T*^ completely specifies a conformation for a molecule with *n* rotational degrees-of-freedom.

**Fig 1:**
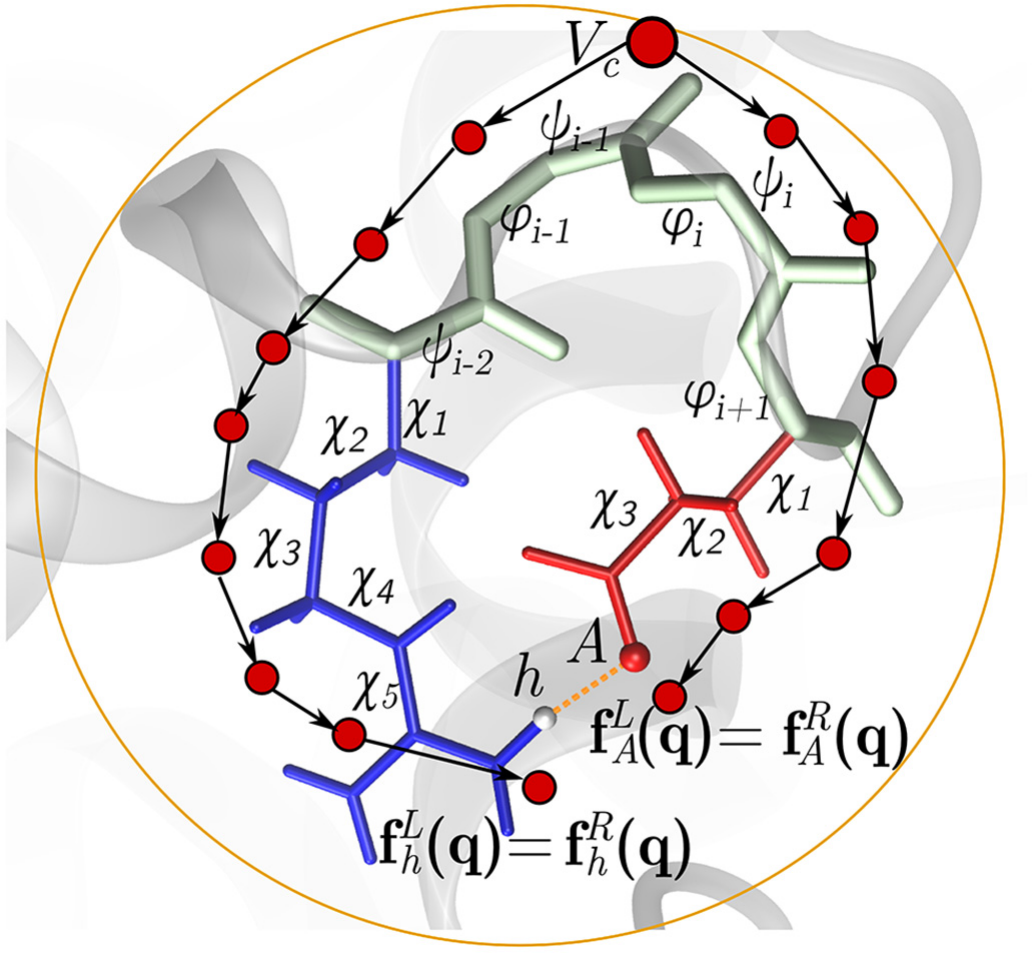
A kinematic representation of proteins. **a)** A protein is represented by an acyclic graph *G* = (*V*, *E*) encoding a kinematic chain. A hydrogen bond *h*–*A* defines a distance constraint, which results in a closed loop. A perturbation to the position of *h* by DoFs along the subtree on the left side needs to be matched by a perturbation to *h* by DoFs on the right side. Similarly for the position of *A*.

We represent the kinematic linkage as a rooted, directed spanning tree, i.e. an acyclic graph *G* = (*V*, *E*) that connects all vertices *V* such that each one, except the root, has only one incoming, directed edge *E*. Vertices *V*_*i*_, *i* = 1, …*B* represent rigid bodies, and edges *E*_*j*_, *j* = 1, …, *n* represent DoFs. Hydrogen bonds are encoded as distance constraints, resulting in closed loops or so-called kinematic cycles in *G* (Fig 1).

### Identifying and perturbing cycles

A cycle-closing hydrogen bond connects two subtrees propagating from a common ancestor rigid body *V*_*c*_ (Fig 1). To avoid hydrogen bond dissociation, a perturbation Δ**q** should leave the positions of the hydrogen bond donor atom *h* and acceptor atom *A* unchanged with respect to a local coordinate frame placed at *A* and *h*.

We denote the DoFs subject to constraints as *cycle* DoFs. For each cycle *i* = 1…*m*, we can define endpoint maps $\text{f}:{𝕊}^{k}\mapsto {\mathbb{R}}^{3},{\text{x}}_{h,A}^{L,R}={\text{f}}_{h,A}^{L,R}(\text{q})$, which map the *n*_*cycle*_ DoFs of the molecular conformation **q** to the hydrogen bond acceptor *A* and donor *h* positions **x**_*h*, *A*_, along the left (*L*) or right (*R*) sub trees stemming from *V*_*c*_. The six holonomic closure constraints

$$\begin{array}{l} f_{h}^{L}\left(q\right)-f_{h}^{R}\left(q\right)=0,\\ f_{A}^{L}\left(q\right)-f_{A}^{R}\left(q\right)=0 \end{array}$$ (1)

define a constraint manifold 𝓜, which is in general (*n*_*cycle*_ − 5*m*)-dimensional. Motions on 𝓜 result in coordinated changes to DoFs that satisfy the distance constraints, and thus maintain hydrogen bonds. However, such motions are difficult to calculate since the constraint manifold is complex. We approximate the manifold locally by its tangent space *T*_**q**_𝓜 at **q**. Differentiating Eq (1) yields

$$\begin{array}{l} \frac{d}{dt}\left(f_{h}^{L}\left(q\right)-f_{h}^{R}\left(q\right)\right)=\left(\frac{df_{h}^{L}}{dq}-\frac{df_{h}^{R}}{dq}\right)\dot{q}=0,\\ \frac{d}{dt}\left(f_{A}^{L}\left(q\right)-f_{A}^{R}\left(q\right)\right)=\left(\frac{df_{A}^{L}}{dq}-\frac{df_{A}^{R}}{dq}\right)\dot{q}=0, \end{array}$$ (2)

which we can rewrite as $\text{J}\dot{\text{q}}=\text{0}$. The 6*m* × *n*_*cycle*_ Jacobian matrix, **J**, gives the instantaneous relationship between the cycle degrees of freedom and the end-point positions and orientations. Entries of the Jacobian matrix are efficiently computed as

$$J_{\text{ij}}=u_{j}\times (r-r_{O_{j}}),$$

where **u** is a unit vector along DoF *j*, **r** denotes the position of the donor or acceptor atom of the cycle-closing bond, and **r**_*O*_*j*__ denotes the position of the tail atom of DoF *j*.

Perturbing a molecular conformation with any vector selected from a sufficiently small neighborhood of the origin in the null-space of **J**, i.e. Ker(**J**) = {**q** ∈ 𝕊^*n*^:**Jq** = **0**} will maintain hydrogen-bond distances. The right-singular vectors of the singular value decomposition **J** = **U** **Σ** **V** ^T^ form a basis, **N**, of the null-space of the Jacobian. Note that **N** is orthonormal, and that **NN** ^T^ is the orthogonal projection onto Ker(**J**). A null-space perturbation projects a trial-vector Δ**q** onto the null-space, Δ**q**_*T*_**q**_𝓜_ = **NN** ^T^Δ**q**.

Previous sampling-with-constraint procedures relied on an elegant combinatorial pebble game algorithm [17] to identify exactly all rigid and flexible substructures in the molecule [15, 24]. The pebble game algorithm, originally developed for 2D network glasses and later validated for 3D molecular graphs by the molecular conjecture [25], explicitly counts constraints and degrees of freedom. Our projection method does not require constraint counting, recognizing that the subset of rigidified degrees of freedom **V** ^*rigid*^ span the nullspace of the projection matrix Ker(**NN** ^T^) in our method:

$$V^{\text{rigid}}=\left\{q\;:{\mathrm{NN}}^{\text{T}}q=0\right\}$$

Note that Ker(**NN** ^T^) never requires explicit computation in our method. Mapping a trial move Δ**q** onto Ker(**J**) by **NN** ^T^Δ**q** naturally projects out the rigidified DoFs.

In addition to *cycle* DoFs, proteins generally have *free* DoFs that are not part of any closed loop and, therefore, not subject to constraints. Note that the designation free or cycle DoF is independent of the choice of the root *R*.

### Conformational energy

Bond lengths and angles are assumed fixed in our kinematic representation, representing bonded energy terms. Non-bonded van der Waals interactions are represented by a hard-sphere, repulsive potential that is scaled for each atom type. We use an efficient grid-indexing method for detecting clashes [26]. While no explicit dihedral energy term is present, disallowed dihedral combinations are avoided by clashes.

### Validation

To validate our algorithm, we selected the three proteins with the largest RMSD between apo and holo conformations from a data set curated for predicting apo conformations from holo conformations [27]. Hydrogen bonds shared between apo and holo conformations were included as constraints. The domains were determined as follows: L-Leucine binding protein (leub) domain 1 residues 1–119 and 251–327, domain 2 residues 120–250 and 328–345; Osmo protection protein (osmo) domain 1 residues 6–109 and 213–275, domain 2 110–212. Alginate binding protein (algi) domain 1 residues 1–133 and 310–400, domain 2 residues 134–309 and 401–490. For each holo conformation, 20,000 random samples were generated with exploration radius of 8Å for leub, 6Å for osmo and 10Å for algi, see the section KGS sampling below for details. To analyze the results, the centers of mass of the holo domains were first aligned with the *z*-axis of the laboratory coordinate system. Domain 1 in each sample in the conformational ensemble was aligned with domain 1 of the holo conformation before the zenith and azimuth angle of domain 2 of the sample were calculated [28].

We used the ligand-free (PDB 2ZIJ) and bound (PDB 1BB5) crystal structures of human lysozyme as starting conformations. We made the L96A mutation to the bound structure to match the wild-type sequence of the ligand-free conformation [29]. Hydrogen bonds shared between the starting conformations were included as constraints. We generated 20,000 random samples with an exploration radius of 4Å. To analyze the results, ensemble conformations were aligned to the backbone heavy atoms of the bound structure. The breathing angle *θ* was computed from the centers of mass of the CA atoms from three protein regions [29]. The RMSD of the CA atoms of secondary structure elements from the bound structure was computed for each ensemble [29]. The angle *θ* and RMSD were binned in 0.5 degrees and 0.1Å to calculate ‘free-energy’ landscapes of these reaction coordinates.

### Hydrogen bonds

KGS takes as input a constraint file, which allows users to identify which distance constraints to maintain. In this study, hydrogen bonds belonging to our modeled Linkers I and II were removed. In both systems, the intersection of the sets of hydrogen bonds for active and inactive states was retained. Eventually, KGS sampling of both states was performed with 130 hydrogen bonds in total.

### System preparation

**Fig 2:**
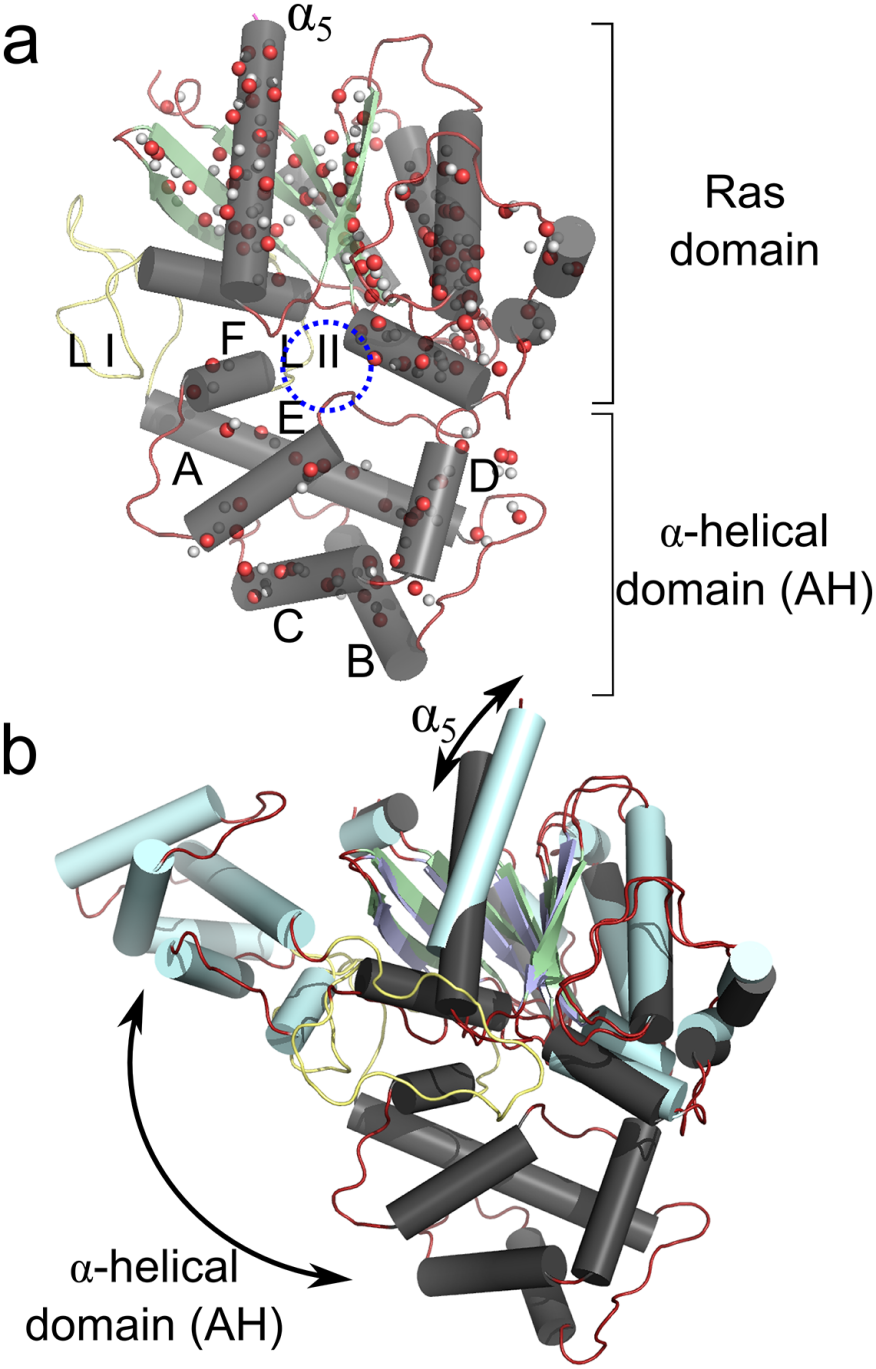
Architecture of G*α*s. **a)** The G*α*s subunit consists of a Ras-domain and an *α*-helical domain. Linker I and II are shown in yellow, and the binding site of the nucleotide in between the two domains is indicated by a blue circle. The location of donor and acceptor atoms of the hydrogen bonding network used for KGS are indicated with white (donors) and red (acceptor) spheres. **b)** The activated state of Gs (pale cyan) superimposed onto the inactive state. Upon activation, the *α*-helical domain undergoes a large rotational motion. Helix *α*_5_ translates and rotates upward to interact with the cytoplasmic core of the receptor.

A structural representative for activated apo G*α*s was extracted from the crystal structure of *β*_2_AR:Gs complex with PDB id 3sn6 [30] and inactive apo G*α*s was obtained from 1azt [31]. The crystal structure of the inactive state of G*α*s had three residue gaps: 1 − 34, 70 − 86, 391 − 402. Residues 70 − 86 (Linker I, Fig 2) were added by Xpleo [16] and subsequently refined in Coot [32]. Finally, the structure was truncated to include residues 35 to 391 (357 residues). The crystal structure of active G*α*s had four residue gaps: 1 − 8, 60 − 87, 203 − 204, 256 − 262. Residues 60 − 87 were built by Xpleo, 203 − 204 were added in Coot, 254 − 265 were copied from the inactive structure of G*α*s after alignment, and the sequence was also truncated to include residues from 35 to 391.

The *α* subunit of Gs consists of a Ras-like domain and an *α*-helical (AH) domain (Fig 2). The Ras-domain is about 260 residues, which is connected to the AH-domain (about 112 residues) by two linkers. The long *α*_1_–*α*_*A*_ linker I, spans residues 65 to 88, and a shorter *α*_*F*_–*β*_2_ linker II spans residues 200 to 206 (Fig 2).

These structures were then parametrized by the CHARMM27 all-atom force field [33] including the CMAP correction [34] and solvated in an octahedral unit cell with 19,737 TIP3 water molecules and electrostatically neutralized by 22 Na and 12 Cl ions (concentration of 0.05 M and no ions within 6Å of any protein atom) for a total of 65,000 atoms. The resulting system was minimized with Gromacs 4.6.3 [35, 36] by a series of steepest descent and conjugate gradient algorithms by gradually reducing constraints on the protein atoms. The minimized structures of active and inactive apo G*α*s served as the input models for the sampling algorithms.

### KGS sampling

The Gs *α* subunit was represented by 1769 rigid bodies and 1768 directed edges corresponding to the dihedral DoFs *ϕ*, *ψ*, and *χ*_*i*_. While any rigid body in the molecule can serve as the root *R*, we set *R* as the first rigid body at the N-terminus of the molecule. There were 767 cycle DoFs in the system. To ensure rapid and broad diffusion of the sampled ensemble, the sampling protocol inspired by Rapidly-exploring Random Trees of previous work was used [14, 15, 37], which we briefly summarize. The sampling pool was initialized with the minimized conformations of active or inactive apo G*α*s **q**_*init*_. We generated a pool of 20,000 samples in an exploration sphere of fixed radius (20Å RMSD) from **q**_*init*_, which was subdivided into shells 𝓢_*i*_, *i* ∈ {1, …, 100} of width 0.2Å, as follows. At each sampling step, a shell 𝓢_*k*_ was selected at random from the subset of shells containing at least one conformation. Next, an entirely random conformation **q**_*random*_ was generated. The conformation that was RMSD-closest to **q**_*random*_ in 𝓢_*k*_ was selected as **q**_*seed*_, and **q**_*random*_ was discarded. A random perturbation Δ**q** to **q**_*seed*_ was proposed, that was then projected onto the constraint manifold and applied to **q**_*seed*_ to obtain a new conformation **q**_*new*_, i.e. **q**_*new*_ = **q**_*seed*_+**NN** ^*T*^Δ**q**. If **q**_*new*_ did not contain clashes, it was added to the pool in the shell corresponding to its RMSD from **q**_*init*_, else it was discarded. The exploration radius and shell width are adjustable parameters. The combination of values selected for this study were found to balance broad exploration and uniform coverage. The collision factor that scales VdW radii during collision detection was set to 0.75. The maximum rotation of a DoF was scaled to 0.29 degrees, which was found to reflect a good balance between fast divergence from initial structure and a high acceptance ratio. To test if the sampling trajectories had converged, we additionally generated a conformational distribution of 50,000 samples around the inactive and active states. All analyses are based on 20,000 samples, unless otherwise stated.

### iMC sampling

We carried out ENM normal modes vibrational analysis (NMA) in internal coordinates (IC) with the software package iMOD [23]. After first obtaining the IC normal modes for each structure with the iMODE tool, we generated a conformational ensemble of 20,000 samples with the default NMA Monte Carlo sampling procedure enabled by the iMC module [23]. We obtained the first 20 normal modes by using all default settings, except enabling *χ* dihedral angles as DoFs to better agree with the KGS DoFs. By default iMC selects from the 5 lowest frequency modes for a Monte Carlo step. S2 Fig. displays all the modes. We used coarse-grained all heavy-atom representation and a sigmoid function pairwise interaction potential with default parameterization. We scaled the parameter *a* (’linear factor to scale motion’) ten-fold to better match the amplitude of domain motions suggested by experimental measurements. Further increasing the scaling did not lead to better agreement.

To examine if a sigmoid function potential possibly over-constrained the system, we also sampled using a coarse-grained, CA-only representation with an essential dynamics (ED) potential function. A scale factor of *a* = 10 agreed with experimental data, but led to distortions in the models. (S3 Fig).

Thus, to enable a direct, one-to-one comparison between KGS and ENM, we selected an all-heavy atom, sigmoid function representation for iMC with amplitudes scaled by *a* = 10, notwithstanding its slightly overconstrained model.

### CONCOORD sampling

We additionally generated conformational ensembles with the distance-restraint based sampling procedure CONCOORD [38]. We used the default, heavy-atom CONCOORD settings for structure and distance bounds generation with OPLS-AA parameters. We used near-default parameters for sampling, using the following command line: disco -on disco.pdb -n 20000 -i 2500 -viol 1. -bump.

### Implementation details and availability

The software is implemented in C++. Calculations were performed on a single, 2.6GHz Intel processor core. Average time to obtain an accepted conformation was 8.9s, at an average acceptance ratio of 30%. Depending on the size of the molecule, computations take from several hours to a few days. No attempts were yet made to optimize the code. The performance limiting step is currently the repeated (𝓞(*n* ^2^)) calculation of RMSD within shells to ensure broad sampling. The shell width balances performance with broad diffusion. The RMSD calculation would be trivially replaced by more modern algorithms that are two orders of magnitude faster [39]. Our SVD calculation is optionally GPU-accelerated. The software and sampling trajectories are available from http://smb.slac.stanford.edu/~vdbedem.

## Results

We first validated our approach of abandoning explicit constraint counting with protein structures determined in different conformations. We then used the KGS, iMC, and CONCOORD ensembles to compare the conformational variability of the *α*-subunit of free, apo G-protein (G*α*s). We examined the ensembles to identify features of the motion associated with the release of GDP and activation of G*α*s.

### Validation

**Fig 3:**
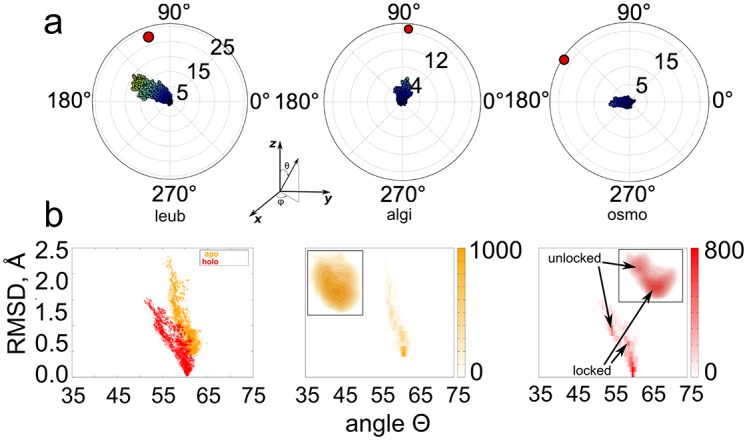
Sampling trajectories on the constraint manifold encode collective motions. **a)** KGS conformational distributions starting from three ligand-free holo crystal structures (leub, algi, osmo) are biased toward the apo structures. The polar plots show the distribution of the angles *θ* (along the radius), and *ϕ* (along the circumference) of the center of mass of domain two with respect to domain one (see Methods). The domains open, reorient, and deform upon adopting the apo conformation (red circle), affecting the relative position of the centers of mass of the domains. The orientation of domain two starts out at the origin. The colors of samples are red-shifted toward higher sampling number. The conformations diffuse toward the apo state as sampling progresses. **b)** The KGS conformational distribution along the reaction coordinates *θ* and RMSD (in Å) to the holo crystal structure of apo and holo human lysozyme. The holo distribution samples more broadly, and more towards smaller *θ* angles than the apo distribution, in agreement with the free-energy landscape observed from RDC restrained simulations (left panel). The middle and right panel show the sampling distribution for apo and holo in more detail. (Inset: free-energy landscapes from RDC restrained MD simulations. Images adapted from [29]). Weak local maxima approximately corresponding to the ‘unlocked’ and ‘locked’ state can be observed in the distribution starting from the holo structure.

To validate our algorithm, we computed conformational distributions for three two-domain protein crystal structures that were determined in both holo and apo conformations. For each protein, the domains open, re-orient and conformationally adjust upon adopting the apo conformation. We observed conformational distributions directed along holo-apo pathways. Starting from the holo conformation, we found that conformational ensembles on the constraint manifold defined by interconnected cycles were highly biased toward the apo conformation (Fig 3a). Polar plots of the distribution of zenith (*θ*) and azimuth (*ϕ*) angles of relative positions of the centers of mass of the two domains reveal domain opening and collective, reorientating motions toward the apo conformation. No conformational pathways connecting the holo substate to the apo substate were observed, but it is unknown if ligand-free holo-apo conformational interconversion occurs for these proteins in solution. Additionally, sampling limitations or steric barriers between the states can prevent end-to-end pathways. Reaching sparsely populated, ‘excited’ substates often demand additional (experimental) restraints on conformational sampling techniques [14, 27, 29].

We furthermore tested whether conformational distributions owing to collective motions on the constraint manifold can accord with free energy landscapes observed in solution. Apo human lysozyme displays large breathing motions, characterized by the angle *θ* between the *α* and *β* domains. The free-energy landscape for the reaction coordinates *θ* and RMSD to the holo crystal structure of apo and holo (triNAG-bound) human lysozyme was recently characterized from replica-averaged, RDC-restrained molecular dynamics simulations [29]. While the free energy of apo lysozyme has a single minimum, the holo state revealed a second, sparsely populated ‘unlocked’ state centered on (49°,1.5Å) in addition to the main ‘locked’ state around (58°,0.9Å) (Fig 3b). The holo protein is capable of sampling a wider range of *θ* angles than the apo structure, presumably to facilitate product release. Our conformational distributions starting from the (ligand-free) holo and apo structures revealed surprisingly similar conformational distributions compared to those from RDC-restrained simulations (Fig 3b, left panel). The holo distribution samples more broadly, and more towards closed conformations (smaller *θ* angles) than the apo distribution, in agreement with the free-energy landscape observed from RDC restrained simulations. Additionally, weak local maxima were observed in the holo distribution, corresponding to the ‘locked’ and ‘unlocked’ state (Fig 3b, right panel). The unlocked state corresponds to a sparsely populated, intermediate state, which was validated experimentally. Thus, collective motions on the constraint manifold enable quick diffusion away from the initial state along biologically-relevant directions that map the conformational landscape of the protein.

### Heterotrimeric G proteins

*β*_2_AR can form a complex with heterotrimeric stimulatory protein G*α*s*βγ*[40]. While the precise mechanism of protein Gs-activation remains poorly understood, interaction with the activated receptor is incidental with the dissociation of GDP and the *βγ* subunits [41]. G*α*s, which binds GTP after the release of GDP, subsequently interacts with many effector proteins in the cell. It is hypothesized that its profound conformational flexibility plays a crucial role in signal modulation [42].

The activated (nucleotide-free) state of G*α*s involves a large motion of the AH-domain with respect to the stable Ras-domain [43]. Additionally, the *α*_5_-helix of G*α*s translates and rotates upward to interact with the cytoplasmic core of the receptor. The domain opening purportedly facilitates the release of GDP. The *β*_6_–*α*_5_ loop, which binds the purine ring of GDP, and the *β*_6_ strand also change conformation (Fig 2). The large distance separating the crystal structures of the active and inactive states suggests that G*α*s can access many different conformations [2, 42, 44]. However, structurally characterizing and determining the sequence of events in the activation pathway by experimental means has proved challenging. Simulations suggest a coupled motion between the AH-domain and helix *α*_5_[13, 45]. Additionally, the opening angle of the AH-domain upon activation is the subject of intense debate. Several lines of evidence suggest that crystal lattice formation may have played a role in selecting an extreme opening angle for the AH-domain [10, 44]. Distance distributions obtained in solution indicate that the conformational variability of the AH-domain of Gi protein in complex with rhodopsin is more limited than that observed in the crystal structure of *β*_2_AR:Gs [10].

### KGS samples the activation pathway of G*α*s

**Fig 4:**
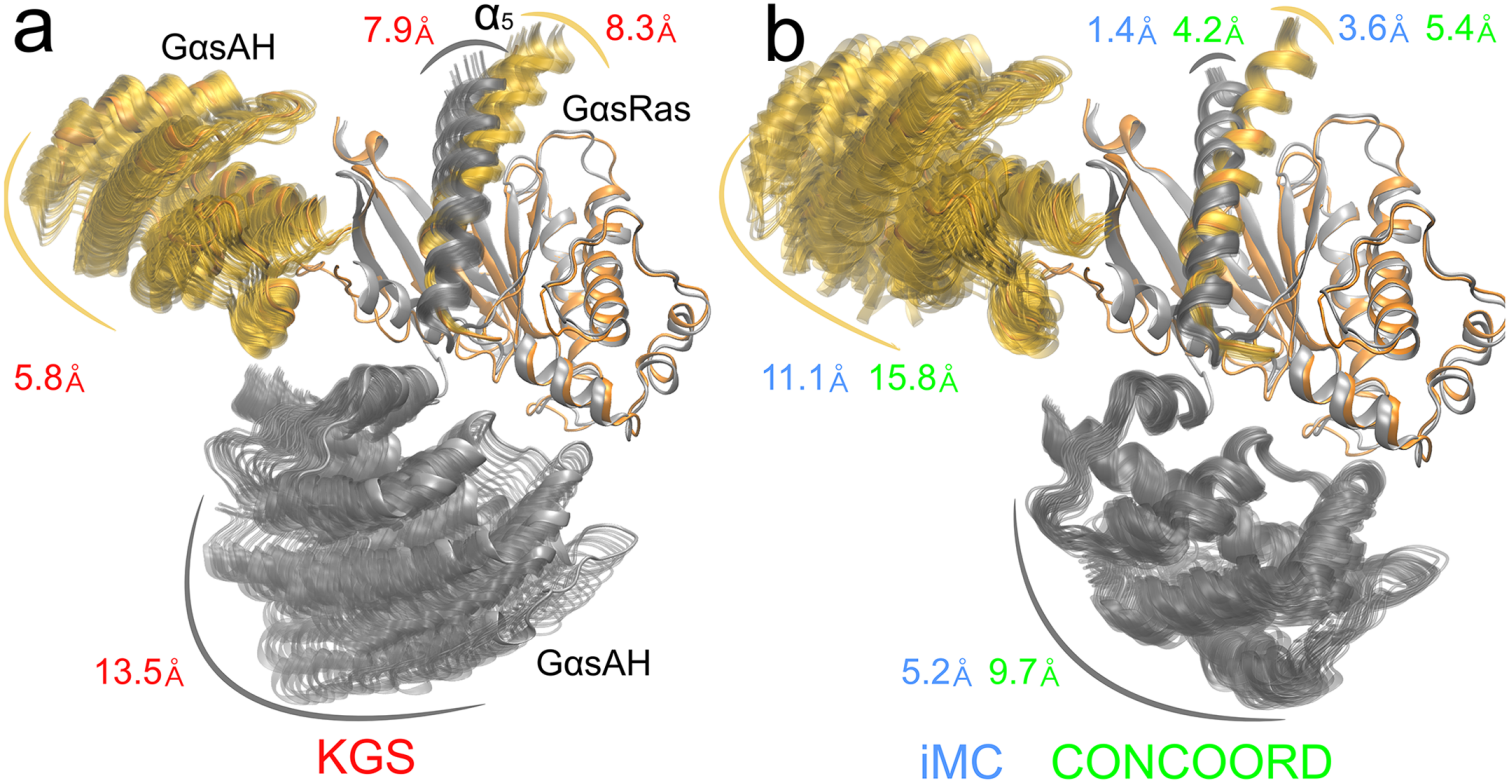
Comparison of the KGS, iMC and CONCOORD active and inactive Gs ensembles. **a)** Conformational ensembles of free, apo G*α*s protein obtained from KGS sampling starting from the active (orange) and inactive (gray) states. Spread of sampling over 20,000 samples is shown for the G*α*s AH-domain and the *α*_5_-helix of the G*α*s Ras-domain. **b)** The same as a), but for iMC and CONCOORD sampling. For visualization, we superimposed the G*α*s Ras-domains within the ensembles.

**Fig 5:**
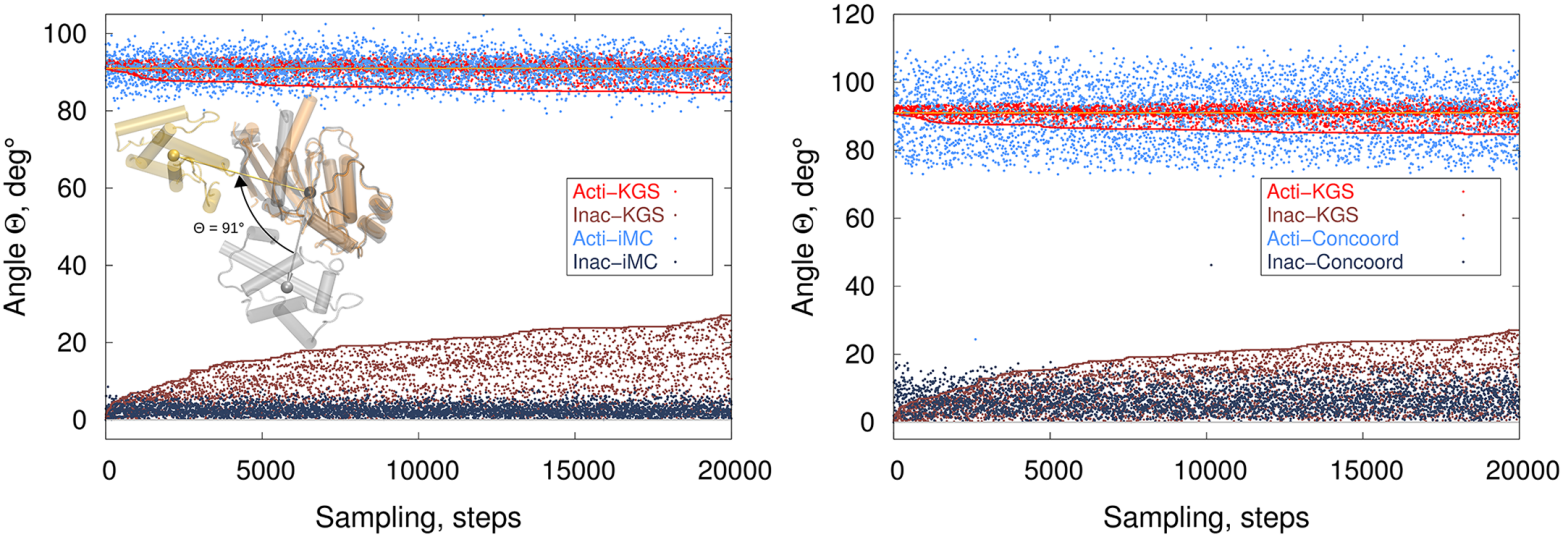
Diffusion of the G*α*s AH-domain opening angle in the ensemble. The change in opening angle between the AH-domain and the Ras-domain is shown as sampling progresses for KGS, iMC (left panel), and CONCOORD (right panel). For KGS, the inactive AH-domain opens to nearly 30 degrees (dark-red), while the active AH-domain ranges from 84.8 to 96.5 degrees (red). The inactive opening angle of iMC reaches 9.9 degrees, and ranges from 79.6 to 103.7 degrees for the active AH-domain. The opening angle of activated G*α*s in the *β*_2_AR:G*α*s crystal structure is indicated in the inset. The inactive opening angle of CONCOORD reaches around 18 degrees, and ranges from 74.6 to 111.4 degrees for the active AH-domain. For the active state, iMC and CONCOORD sample uniformly around the starting angle. The angle of the KGS samples slowly decreases towards the inactive state.

We first examined the conformational variability of the AH-domain between the active and inactive states with the methods KGS, iMC, and CONCOORD. The RMS deviations for KGS samples starting from the inactive state of the AH-domain of G*α*s was 13.5Å, while for the active state it was 5.8Å (Fig 4a). For iMC, the observed values were 5.2Å and 11.1Å, and for CONCOORD 9.7Å and 15.8Å (Fig 4b). In addition, all three methods identify large motions of helix *α*_5_ concurrent with the domain motions. The maximum opening angle Θ_*max*_ between the two domains was 27.2 degrees (Fig 5 and S4 Fig, 37.9 degrees for 50,000 samples) for the inactive state KGS ensemble, compared to 91 degrees for the activated crystal structure (Fig 2). iMC reported a maximum opening angle for the inactive AH-domain of around 9.9 degrees (Fig 5, left panel). The CONCOORD conformational ensemble reported a range of 15 − 20 degrees of an opening angle around the active state, and 18 degrees around the inactive state (Fig 5, right panel).

Both iMC and CONCOORD sample with nearly uniformly fixed radius around the active starting conformation, which is rationalized by their reliance on an equilibrium conformation (Fig 5). In contrast, KGS, by design of its RRT-based sampling avoiding steric collisions, mimics a trajectory diffusing out of the starting conformation. KGS sampling of the activated state exhibited a slowing rate of change, while the opening angle of the inactive state still appeared to increase slightly at 20,000 samples, leveling of at 50,000 samples (S4 Fig). The lack of full convergence did not appreciably change the conformational distributions, but can moderately limit interpreting the ensemble as a collection of exchanging conformational substates. Interestingly, while KGS sampling of the active conformation initially exhibits greater conformational diversity away from the inactive state, later samples are directed more towards the inactive state.

The KGS ensemble for free, apo G*α*s compares very well with the RMSD and opening angle reported from experimental observations in solution. Double Electron-Electron Resonance (DEER) spectroscopy measurements suggest that the average displacement of the apo AH-domain of Gi protein complexed with rhodopsin is 15Å [10]. From the nine models of receptor-bound Gi conformations reporting on the DEER observations, we measured an equivalent average opening angle Θ of 25.5 degrees (Θ_*max*_ = 48.8 degrees) after alignment to the G*α*s Ras-domain.

The KGS ensemble suggests that ligand-free G*α*s is structurally and evolutionary designed to access a broad range of opening angles. However, a set of discrete samples connecting the inactive with the active state of G*α*s (Fig 4a and 4b) was not observed. The sample acceptance ratio in KGS, i.e. samples not rejected owing to collisions between atoms, also differed substantially between the inactive and active states (35% vs 15%). These findings could signify a steep conformational barrier between the inactive and active crystal structures between 40 to 80 degrees of domain opening angle. For instance, in the activated state of *β*_2_AR:G*α*s, the *α*_1_-helix of the Ras-domain is partially melted to accommodate the large motion.

### Directionality of displacement

**Fig 6:**
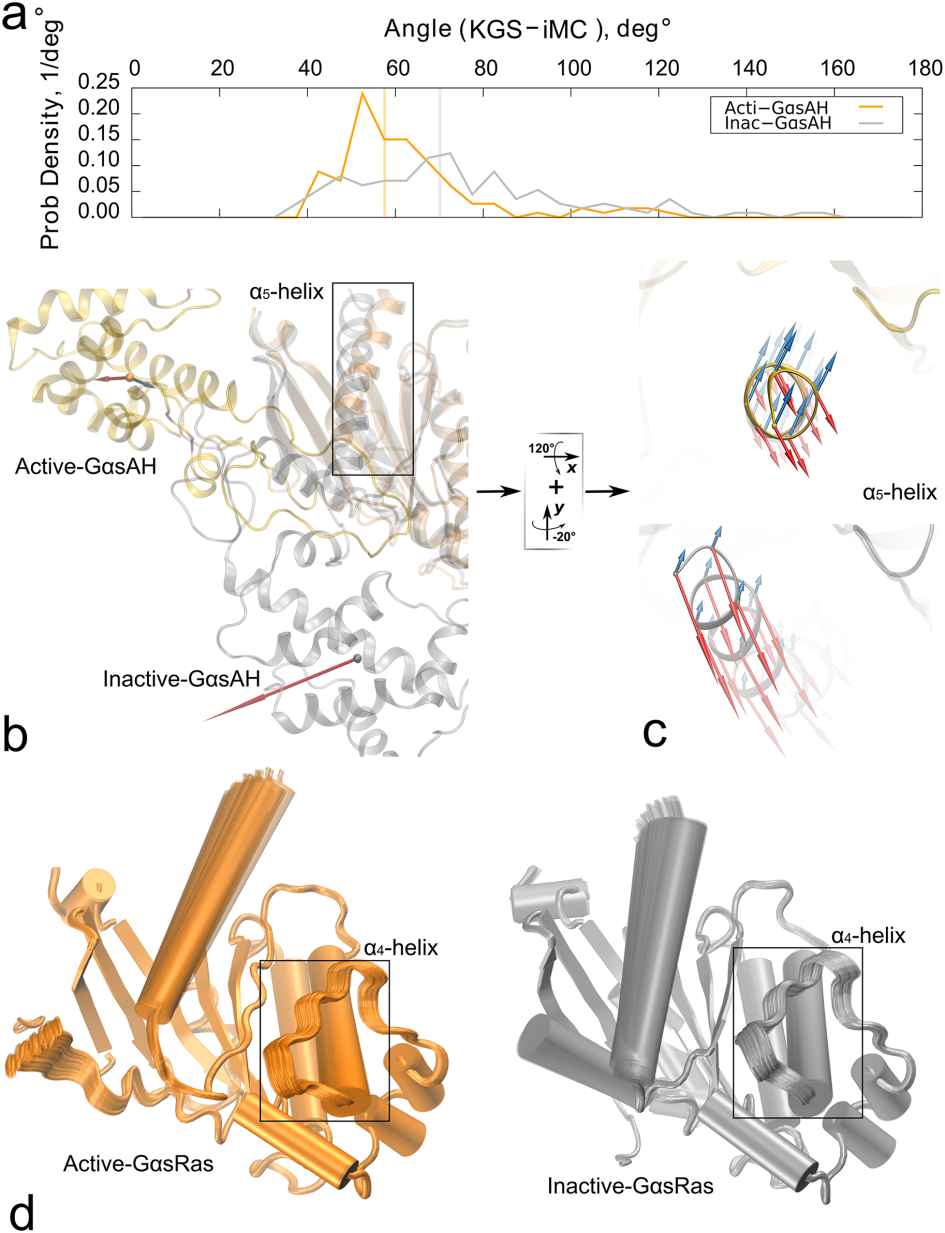
Direction of displacement of the AH-domain. **a)** Relative frequency of the angles between KGS and iMC average displacements for each C_*α*_ of the AH-domain in the inactive (grey) and active states (orange/yellow). The average AH-domain displacement is shifted by 50 to 60 degrees for the states between the two methods. **b)** Differences in the directions of the mean displacement of the center of geometry of the G*α* AH-domain between the KGS (red) and iMC (blue) ensembles. **c)** Directionality of the CA displacements of the *α*_5_-helix in the KGS (red) and iMC (blue) ensembles in the active state (top) and inactive state (bottom). The motion in the KGS ensemble is directed from the inactive conformation of *α*_5_ to the active conformation. d) Superimposed Ras-domains from the KGS ensembles for the active state (yellow) and inactive (grey) states. The amplitude of the Ras-domain ensemble is limited, except for marked fluctuations of helices *α*_4_ and *α*_5_.

In the remainder, we focus on a direct comparison of the directional conformational variability of KGS and iMC since these methods are conceptually most alike. We calculated a distribution of angles between the mean displacements of C_*α*_ atoms of the G*α* AH-domain for the two ensembles (Fig 6a and S5 Fig). The mean displacement is the vector connecting the center of mass of all AH-domain C_*α*_ atoms of the initial structure to the averaged center of mass of the ensemble, after alignment to the stable part of the Ras-domain. The angles of mean displacement for the AH-domain are visualized in Fig 6. The angles of mean displacements did not align but were significantly shifted for both the active (57.6 degrees, Fig 6a yellow bar) and inactive state (70.2 degrees, Fig 6a gray bar). The long tails for the angle distributions, in particular for the inactive state, identify a significant number of residues for which the angle differ by more than 90 degrees. Thus, large-amplitude motions of G*α*s are described differently by the two procedures.

The conformational distributions from KGS starting from the inactive form of G*α*s aligns with the proposed activation mechanism of the *β*_2_AR:G*α*s after GDP release. The direction of motion for the KGS inactive ensemble corresponds to a domain opening motion in the viewing plane, with a small component orthogonal to the viewing plane (Fig 6b). The iMC motion is nearly orthogonal to the viewing plane, resulting in a transverse ‘rocking’ motion, with a moderate component downward towards a domain opening motion. Floquet and coworkers observed a similar, pivoting motion for the AH domain around the *α*A helix, which is implicated in GDP release, from Cartesian NMA with the CHARMM27 force field for protein Gi [13, 46].

The size of the vectors reflects the difference in RMSD amplitude of the ensembles. For the active state both methods have a significant component orthogonal to the viewing plane. For neither method the main displacement in the active state appears to be along the activation pathway, signifying that additional mechanisms, such as GTP hydrolysis, likely play a key role in G*α*s. The direction of mean displacement for iMC is nearly identical for the inactive and active ensembles. A possible explanation is that local structural changes in the AH-domain between the active and inactive state are modest, leaving interactions defined by the ENM largely unchanged between the states.

Receptor-induced conformational changes in helix *α*_5_ are believed to contribute to GDP release [10, 47]. Concomitant with activation, helix *α*_5_ undergoes a rotation and translation towards *β*_6_. The magnitude and direction of these fluctuations in the KGS ensemble are striking, coinciding with those observed in MD simulations [45]. Fig 6c shows the view looking towards the cytoplasm from the receptor core. The top panel shows the *α*_5_ helix in its active conformation, and the bottom panel in its inactive conformation. The distribution of magnitudes and directions of the KGS displacement vectors along the helix in the inactive state (bottom panel) correspond remarkably well to a translation and rotation along a path to reach the active state (top panel). RMS amplitudes of 8.3Å and 7.9 Å were observed for *α*_5_-helix in the KGS ensemble of the active and inactive states. By contrast, the iMC displacement vectors are slightly smaller in magnitude in the inactive state (indicated by RMS spread of 1.4Å and 3.6 Å) and have a component nearly opposite to the activation pathway. Note that while in general normal mode vectors indicate undirected displacement, our displacement vectors were calculated directly from the ensembles.

### KGS displacements highlight collective motion

**Fig 7:**
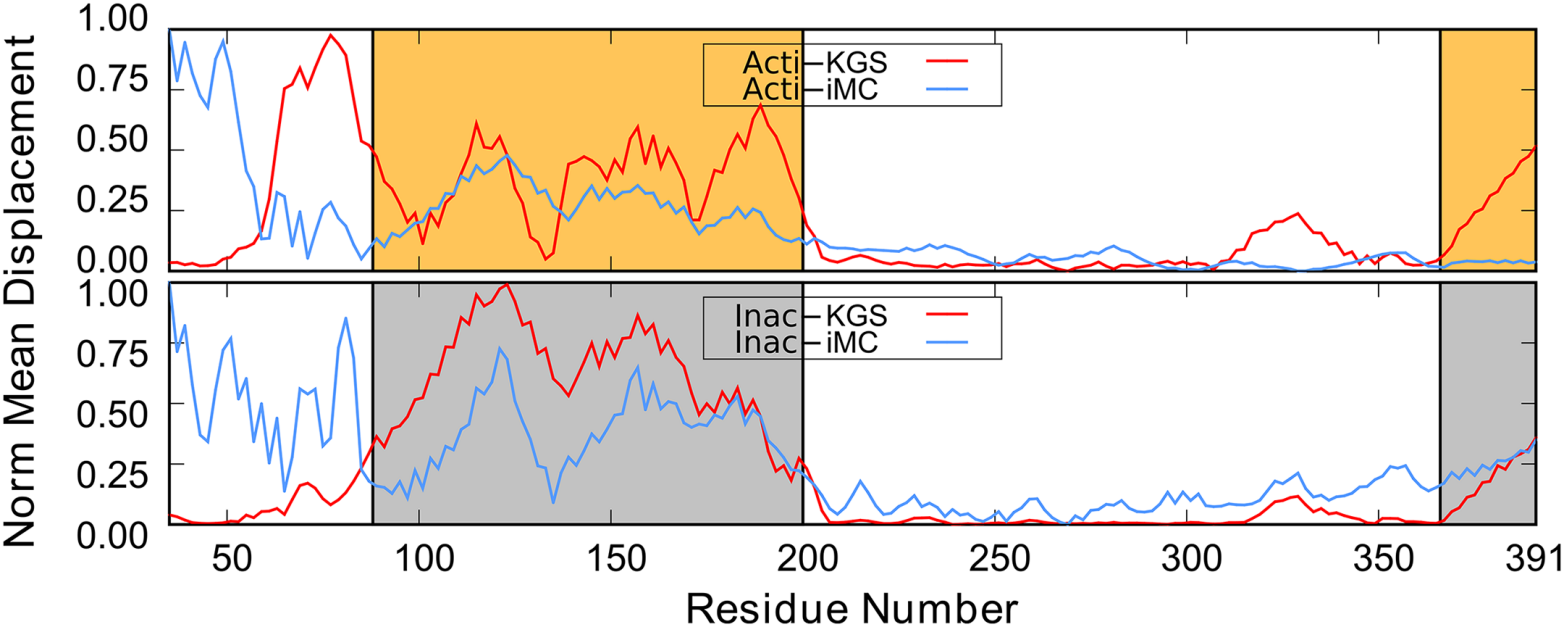
The normalized magnitude of the mean C_*α*_ displacement vectors of the KGS and iMC ensembles. The top panel shows the normalized displacements for the active state KGS and iMC ensembles. The bottom panel for the inactive state. The AH-domain is indicated by orange (active) or grey (inactive) shading. The *α*_5_ helix is shaded in the same color on the far right. The location of the N-terminal part of *α*_4_ helix in the Ras-domain is indicated by a pronounced “bump” in between residues 320 and 340 for the KGS ensemble (red).

Next, we analyzed displacements at the residue level for both domains of G*α*s. Fig 7 (S5 Fig) shows the normalized magnitude of the mean CA atom displacement vectors of the ensembles. Each displacement vector was calculated as the average RMSD vector of all conformations after alignment to the stable part of the Ras-domain (as above), and normalized within the angle values of its own ensemble.

Mean displacements for the KGS and iMC sampled G*α*s ensemble exhibit a clear pattern; they are larger for the AH-domain and vanishingly small for the Ras-domain in both the active and inactive state. The coordinated perturbations of DoFs by KGS resulted in intra-domain displacements shared by groups of contiguous residues. Three regions of the AH-domain separately display collective features indicated by elevated mean displacements, corresponding to the C-terminus of *α*_*A*_ and *α*_*B*_, *α*_*C*_ and *α*_*D*_, and *α*_*E*_ and *α*_*F*_. A remarkably similar pattern is observed for the iMC ensemble. Helices *A* − *D* are located towards the outer radius of the rotation of the AH-domain, explaining the elevated levels of mean displacement in both active and inactive state (Fig 2b). Surprisingly, their relative orientation remains well-preserved despite a sparse inter-secondary structure hydrogen-bond network in the AH-domain. The pattern of displacements is similar for the active and inactive state.

Analysis at the residue level reveals key details suggesting collective motion. In the Ras-domain, helix *α*_5_ shows a large displacement, exceeding the mean displacement values of the Ras-domain (Fig 7, right-most shaded bands). The growth in amplitude towards the C-terminus is characteristic for the rotational motion we observed in the previous section. Interestingly, the single, unique feature standing out in an otherwise flat Ras-domain is elevated displacement for helix *α*_4_ and loop *α*_*G*_–*α*_4_ (residues 320–340) in both active and inactive state (Fig 7). Fig 6d shows the motion of *α*_4_ and the adjacent loop. Helices *α*_*G*_ and *α*_4_ are implicated in GDP release. Similar motions were observed using Cartesian NMA with the CHARMM27 force field [13] in protein Gi. Strikingly, both the *α*_5_ and motions of *α*_4_ and the adjacent loop are absent in the iMC Gs active ensemble, but both are present in KGS. This strongly suggests these motions are conformationally coupled, but possibly shifted to higher modes in iMC.

The mean displacements up to residue number 80 suggest anti-correlated motions in iMC and KGS in the active state (Fig 7, top). The mean displacement reported by iMC is elevated owing to restraints between the BC loop in the AH domain and (truncated) helix *α*_1_. This results in collective motions of the *β*_1_-strand with the highly mobile AH domain. The amplitude of the iMC motions is likely overestimated, as it leads to significant distortions of the *β*-sheet in the Ras domain (S6 Fig). Similarly, the proximity of Linker I to helix F leads to collective motions in iMC. The absence of explicit constraints, i.e, hydrogen bonds in Linker I suppresses collective motions in KGS. While the precise nature of Linker I motions remains unclear, the absence of well-defined electron density in the crystal suggests this loop is highly mobile.

### Conformational coupling related to release of GDP

To examine the origin of collective motion, we analyzed the distribution of the DoFs in the conformational ensembles. We observed key differences between the two methods in the spatial distribution of flexibility throughout the protein. The mean RMSF for free and cycle DoFs are summarized in Table 1.

**Table 1:** Mean KGS and iMC RMSF (measured in degrees) for free and cycle DoFs in the active and inactive state.

	Inactive		Active	
	free	cycle	free	cycle
KGS	1.104	0.514 (0.860)	0.932	0.445 (0.737)
iMC	0.83	1.43	0.90	1.60

**Fig 8:**
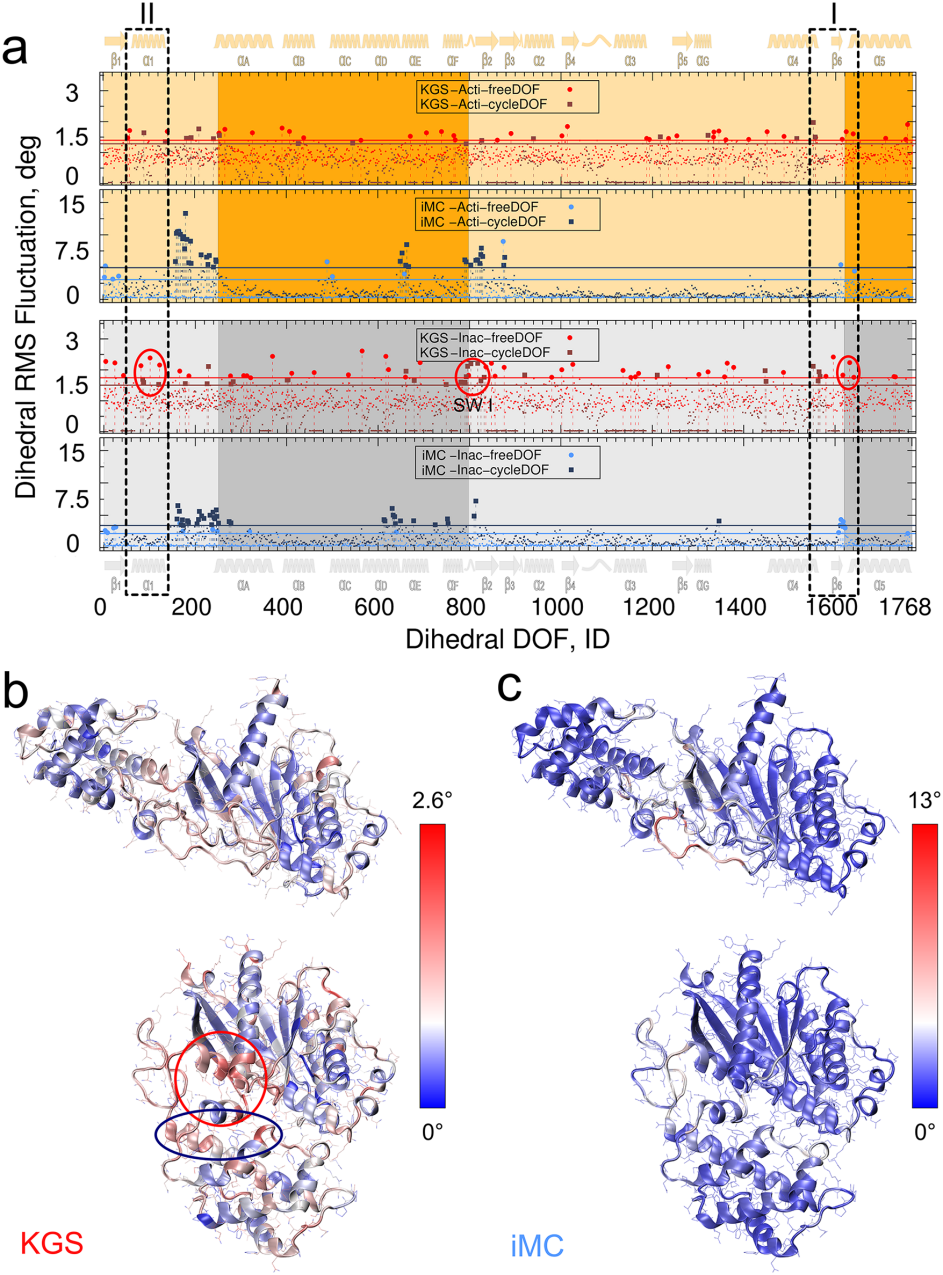
Heatmaps of conformational variability. **a)** RMS fluctuations of DoFs for KGS (red) and iMC (blue) for 20,000 samples starting from the activated conformation (top two panels, orange) and the inactive conformation (bottom two panels, grey). The DoFs corresponding to the AH-domain and helix *α*_5_ are colored in darker shades. Free DoFs are circles, cycle DoFs are squares. The horizontal lines correspond to the mean RMSF value of the DoFs plus 2*σ*. **b,c)** Heatmaps representing the contribution of DoFs to conformational variability for KGS (b) and iMC (c). Coupling in the GDP binding pocket (red circle, *α*_5_, *α*_1_, and the adjacent *β*_1_–*α*_1_ loop (P-loop)) extends to include helix *α*_*F*_ (bottom of the red circle), Linker II (SW I), and the N-terminus of *α*_*E*_ (right side of the blue oval).

Cycle DoFs are uniformly distributed throughout the protein. In KGS, 43.4% of total DoFs are cycle DoFs and of those 41% are rigidified, indicated by vanishing RMSF for cycle DoFs (Fig 8). These DoFs are contained in the null space of the projection matrix **NN** ^*T*^. Rigidified cycle DoFs correspond largely to secondary structure elements, where DoFs are overconstrained by short or overlapping cycles. Free DoFs have larger RMSF than cycle DoFs (Table 1). If rigidified DoFs are excluded from the RMSF, a modest reduction of 20.5% in flexibility from free to cycle DoFs is observed.

By contrast, while iMC does not define free or cycle DoFs, we observed a reversed flexibility trend compared to the corresponding DoFs in KGS–the cycle DoFs are 1.8 times more flexible than free DoFs for iMC. One possible contributing factor to this somewhat paradoxical find is that normal modes are obtained from eigenvectors of the Hessian matrix defined by the potential function. Free DoFs, like those in surface side-chains, are, on average, subject to fewer restraints, and thus less likely to contribute to major modes. The magnitude of a trial move is scaled by the eigenvalues of the modes, and more constrained areas may thus dominate the size of the move. We also observed that large parts of the Ras-domain do not show elevated RMSF with iMC (Fig 8), signifying that many vibrational frequencies rather than a single mode dominate structural changes for this domain.

iMC locates elevated flexibility mainly in loop residues (Fig 8). Linkers I and II stand out, as well as the *β*_6_–*α*_5_ loop. Note that the backbone DoFs for LI and LII are cycle DoFs owing to hydrogen bonds between, for instance, *β*_1_ and *β*_2_. By contrast, elevated variability in KGS is concentrated less in loop areas, and distributed more uniformly throughout the protein.

The magnitude of helix *α*_5_ RMSD spread is nearly identical in the two states. However, small, motional differences in specific helical sub-regions can signify different functionalities. Significant flexibility towards the C-terminal part of the helix would enhance *α*_5_-helix conformational selectivity for inactive-like conformers, while a more active-like conformation would promote specificity through small-scale deformations near the N-terminal part of the helix. For the inactive state we observe elevated variability in the KGS ensemble from the C-terminus of *α*_4_, through *β*_6_, up to the N-terminus of *α*_5_ (Fig 8a, dashed rectangle I). The C-terminus of helix *α*_5_ and the *α*_4_–*β*_6_ loop interact with the receptor. A conserved TCAV motif in the *β*_6_–*α*_5_ loop binds the GDP guanine ring. Helix *α*_5_ and strand *β*_6_ transmit receptor-induced conformational changes to facilitate GDP release [40]. KGS elevated variability is present in the inactive state, but moderated in the activated state and shifted away from the *β*_6_ strand. The magnitude of variability is reduced from inactive to active state for both sampling techniques, suggesting that smaller changes dominate this area in the active state. This interpretation is supported by iMC motions, where elevated variability shifts from *β*_6_ to the N-terminus of *α*_5_ upon activation.

A heat map of conformational changes reveals a hotspot of highly elevated flexibility near the GDP binding pocket in the inactive state (Fig 8b, bottom left circled). Similarities with a heat map obtained from peptide amide hydrogen-deuterium exchange mass spectrometry (DXMS) experiments, which report on exchange rates of amide hydrogens, are striking [48]. While the increased exchange rates established general sensitivity to GDP release, our nucleotide-free analysis suggests that increase in dynamics or disordering of this segment is, at least partly, attributable to motion of helix *α*_5_ and the AH domain.

We also observed conformational coupling of the N-terminus of helix *α*_5_ to *α*_1_, and the adjacent *β*_1_–*α*_1_ loop (P-loop), which binds the nucleotide phosphate (Fig 8a, dashed rectangle II and circled in inactive state). How the elevated flexibility is further coupled is illustrated in Fig 8b, left panels. Coupling in the GDP binding pocket extends to include helix *α*_1_, helix *α*_*F*_, Linker II (SW I), and the N-terminus of *α*_*E*_. Functional, conformational coupling is revealed to a lesser extent by iMC (Fig 7b, right panels). In particular, the close coupling around the GDP binding pocket appears absent, and elevated flexibility is mostly located in loop residues. For iMC, variability of *α*_*E*_ is shifted towards the C-terminal end of helix *α*_*D*_.

## Discussion

Proteins interconvert between functional, often sparsely populated conformational substates at a multitude of spatiotemporal scales to perform their function and interact with other biomolecules [49–51]. Understanding how these substates probe the conformational landscape and how they are coupled through collective motions can provide insights into molecular mechanisms and protein function [52, 53].

Our conformational sampling algorithm maps small random perturbations, highly suggestive of equilibrium fluctuations, onto a constraint manifold that is defined by the hydrogen bonding network. Our new method does not require explicitly calculating rigid substructures of the protein. Instead, DoFs are subject to coordinated motion on the constraint manifold, and DoFs in isostatic or overconstrained substructures are intrinsically rigidified. Cycle DoFs contribute significantly to the distribution of the resulting conformational ensemble. Cycle DoFs make up nearly half of the DoFs, are distributed throughout the molecule, and their RMSF is only moderately reduced compared to free DoFs.

The coordinated motion and distribution of cycle DoFs can potentially provide new information about mechanisms of conformational coupling. Compared to iMC, we observed motions with larger amplitudes, but both methods were in agreement with accepted mechanisms. We were better able to distinguish molecular mechanisms, and locate the origin of conformational flexibility. Important rotational DoFs stand out, and are, surprisingly, located not just in loops to accommodate inter domain motion. Results for Linker I and (activated) loop residues 254–265 should be interpreted with care, as experimental evidence to support their initial conformation is limited. Conformational coupling in the iMC ensemble was less pronounced, and sometimes more difficult to distinguish owing to higher modes or reduced motional amplitudes. The limited range of motion of the iMC sampling procedure likely results from the assumption of harmonic vibrations around equilibrium positions in the ENM. Large deviations break the underlying assumptions and would perturb the topology of the initial conformation–drawbacks that KGS intrinsically avoids. In contrast to the KGS distributions, the RMSD for the inactive state are reduced compared to the active state. The interface between the AH-domain and Ras-domain is subject to restraints imposed by the ENM, which limits the amplitude of the motion along the activation pathway from the inactive state. Nonetheless, while the active state sampled ensemble exhibits a larger RMSD than the inactive state, an overall reduced amplitude with respect to KGS was observed. Interdomain ENM restraints in the direction of the activation pathway alone do not explain the reduced RMSD.

We observed a KGS ensemble along a pathway associated with activation for the *α* sub-unit of protein Gs. Conformational interconversions can occur through a myriad of alternative transition pathways. Computationally probing a multi-state conformational landscape through extensive MD simulations to obtain a probable minimum free energy pathway is often prohibitively expensive. In addition, sampling is generally affected by limitations and imperfections of the force fields [54]. At the expense of highly accurate energy estimates, our method efficiently explores the conformational space accessible to a protein while it maintains exactly covalent and hydrogen bond geometry, and avoids steric clashes. Nonetheless, interpretation of the ensemble as a collection of exchanging conformational substates would require long sampling trajectories to satisfy ergodic properties. Our method illuminates coupled intra- and interdomain motions, complementary to rigid-body domain sampling and subsequent loop rebuilding [10]. Paired with sophisticated MD simulations or energy relaxation protocols [55, 56] our conformational ensemble can, for instance, serve as starting points for detailed transition path sampling.

An exceptionally striking feature of our KGS ensemble is the magnitude of fluctuation of helix *α*_5_ concurrent with the G*α*s AH-domain motion. These coupled motions point to a potential molecular mechanism of *concomitant*, structural changes between two remote sites implicated in the release of GDP upon activation. There is increasing experimental evidence to support this mechanism, which was first predicted by computational means for protein Gi by Floquet and coworkers [13]. Their NMA-based analysis of GDP-bound Gi identified a motion for the AH-domain that pivots on the long axis of the *α*A helix. Surprisingly, this transverse motion qualitatively agrees with our nucleotide-free iMC analysis. The similarity of nucleotide-free and nucleotide-bound motions is likely owed to ENM interactions between the Ras and AH domain in iMC, which mimic interactions of the nucleotide with each domain. Essential Dynamics Analysis (EDA) of AH domain motions upon ejection of GDP on the phosphate side from selected nanosecond time scale Targeted Molecular Dynamics trajectories furthermore revealed close agreement with motions from NMA analysis [46]. The transverse motion likely plays a key role in GDP release and Gi activation at nanosecond time scales. By contrast, whereas the AH domain motion for G*α*s observed from KGS analysis also exhibits the small transverse component, it is mainly directed along a domain opening trajectory in agreement with DEER measurements, potentially additionally identifying longer, micro- to millisecond time-scales motions. While it is speculative to join analyses from two different proteins, these observations do suggest an activation mechanism whereby a transverse ‘rocking’ motion facilitates or results from GDP release, which in turn leads to a domain opening motion. For inactive, apo G*α*s, elevated mobility is centered on a hotspot near the GDP binding pocket, extending to the N-terminus of helix *α*_5_, *α*_1_, and the adjacent P-loop. The mobile helix *α*_4_ is conformationally coupled to the hub through *β*_6_.

Previous studies established that a conformational ensemble obtained by maintaining hydrogen bonds through iteratively refitting rigid substructures agrees well with MD simulations [57]. In our method, the coordinated motions on the constraint manifold resulting from hydrogen bond encode ‘natural’ modes of deformation. However, these coupled motions and broad diffusion by a carefully selected sampling strategy come at the expense of a greatly simplified energy function. It allows our method to overcome high-energy barriers, but can lead to conformations with high physical energies. Thus, care should be taken in interpreting individual ensemble members prior to extensive energy minimization. Hydrogen bonding networks enforce collective motions that couple conformational substates implicated in GDP release. Our results highlight that in addition to stabilizing tertiary structure, hydrogen bonding networks mediate molecular mechanisms and dynamics. Indeed, evidence is emerging that hydrogen bonds mediate longe-range, correlated motions [58].

Our nullspace sampling procedure with explicit, holonomic constraints can relate motion to function by revealing molecular mechanisms. It enables researchers to formulate testable hypotheses about networks of residues that facilitate motions implicated in GDP release and AH-domain motion. In addition, our procedure could be augmented with intra-molecular distance constraints obtained from experimental data.

## Supporting Information

S1 Fig Rigid bodies are the largest groups of atoms without internal rotational degrees of freedom. Five rigid bodies are shown in different colors for a small fragment of a protein. Rotational degrees of freedom (dihedrals) are shown in red. The side-chain of the green rigid body is truncated at the C*β* atom for clarity. A cycle closing hydrogen bond is shown as a thin red line.
(TIFF)

S2 Fig Normal modes for free, apo G*α*s. **a)** The ten lowest frequency normal modes for the active state of G*α*s. **b)** The ten lowest frequency normal modes for the inactive state of G*α*s.
(TIFF)

S3 Fig Domain opening angles for iMC conformational ensembles. The ensembles were obtained from a coarse-grained, CA-only representation with an essential dynamics (ED) potential function. A scale factor of *a* = 10 was applied. The average (25.5 degrees) and maximum (48.8 degrees) opening angles for the AH domain suggested by DEER experiments are indicated by dashed horizontal lines. While the opening angle of the inactive ensemble matches that of the DEER experiment, conformations in the inactive and active ensembles are distorted.
(TIFF)

S4 Fig Diffusion of the G*α*s AH-domain opening angle in the ensemble of 50,000 conformations. **a)** The change in opening angle between the AH-domain and the Ras-domain as sampling progresses for KGS. The maximum opening angle levels around 48,000 samples. **b)** The distributions of the opening angle for the active (top) and inactive (bottom) state from 20,000 and 50,000 samples. The active state distributions are very similar, while the inactive distributions differ in the tail. The active, 50,000 sample distribution has min = 83.9°, mean = 90.6°, and max = 96.6° opening angles. For the inactive distributions these numbers are: min = 0.0°, mean = 14.2°, max = 37.9°.
(TIFF)

S5 Fig Direction and magnitude of normalized mean displacements in KGS and iMC conformational distributions for 50,000 KGS samples. Top panel. The relative frequency of the angles between KGS and iMC average displacements for each C_*α*_ of the AH-domain in the inactive (grey) and active states (orange/yellow) is virtually unchanged in the larger KGS ensemble. Bottom two panels. The normalized magnitude of the mean C_*α*_ displacement vectors of the KGS (50,000 samples) and iMC ensembles. The longer trajectories support stronger coupling for helix *α*_5_, but otherwise are similar to those obtained from 20,000 samples.
(TIFF)

S6 Fig Anti-correlated motions between KGS and iMC at the N-terminus in the active state Ras domain. **a)** The proximity of the BC loop (red, with one residue in stick representation) in the AH domain and N-terminal helix *α*_1_ (with one residue in stick representation) results in ENM restraints for the active state crystal structure. **b)** A snapshot of the iMC conformational ensemble. The *β*_1_ strand (red) is coupled to the AH domain, resulting in large motion amplitudes that deform the *β*-sheet.
(TIFF)
