# Supplementary figures and images for: Nullspace Sampling with Holonomic Constraints Reveals Molecular Mechanisms of Protein Gαs
Source: PLoS Comput Biol. 2015 Jul 28;11(7):e1004361. doi: 10.1371/journal.pcbi.1004361 (PMC4517867; doi:10.1371/journal.pcbi.1004361)

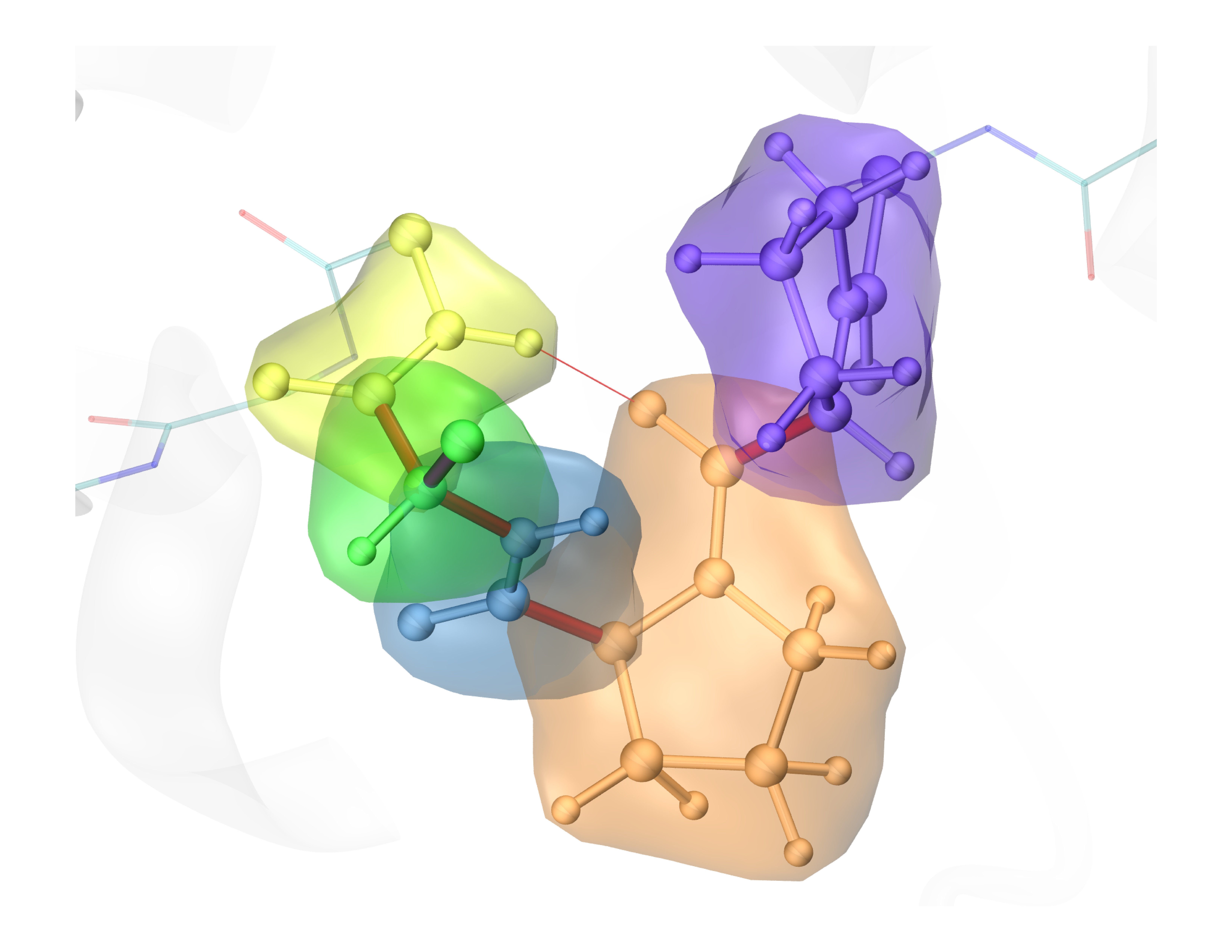

Supplement: S1 Fig — Five rigid bodies are shown in different colors for a small fragment of a protein. Rotational degrees of freedom (dihedrals) are shown in red. The side-chain of the green rigid body is truncated at the Cβ atom for clarity. A cycle closing hydrogen bond is shown as a thin red line. (TIFF) [file pcbi.1004361.s001.tiff]

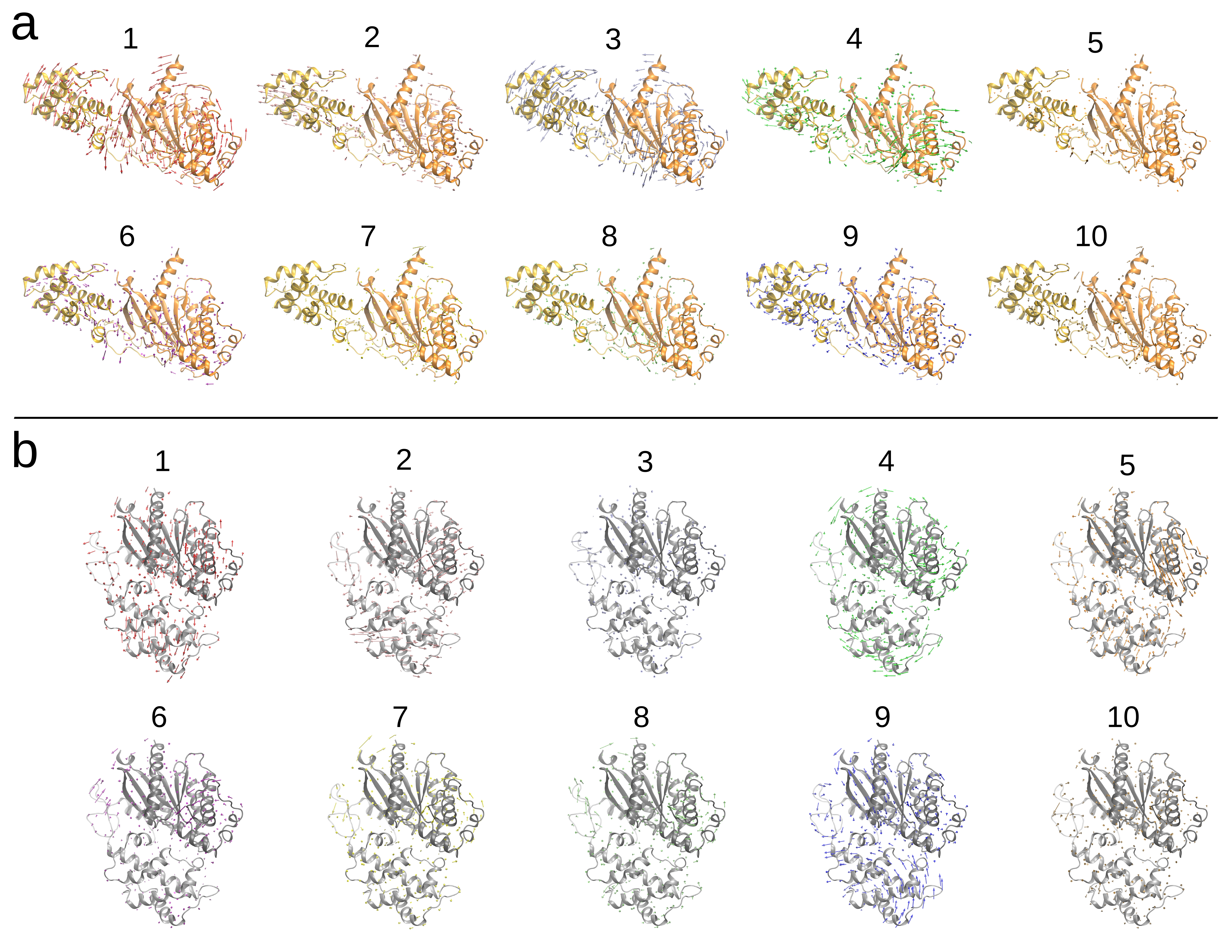

Supplement: S2 Fig — a) The ten lowest frequency normal modes for the active state of Gαs. b) The ten lowest frequency normal modes for the inactive state of Gαs. (TIFF) [file pcbi.1004361.s002.tiff]

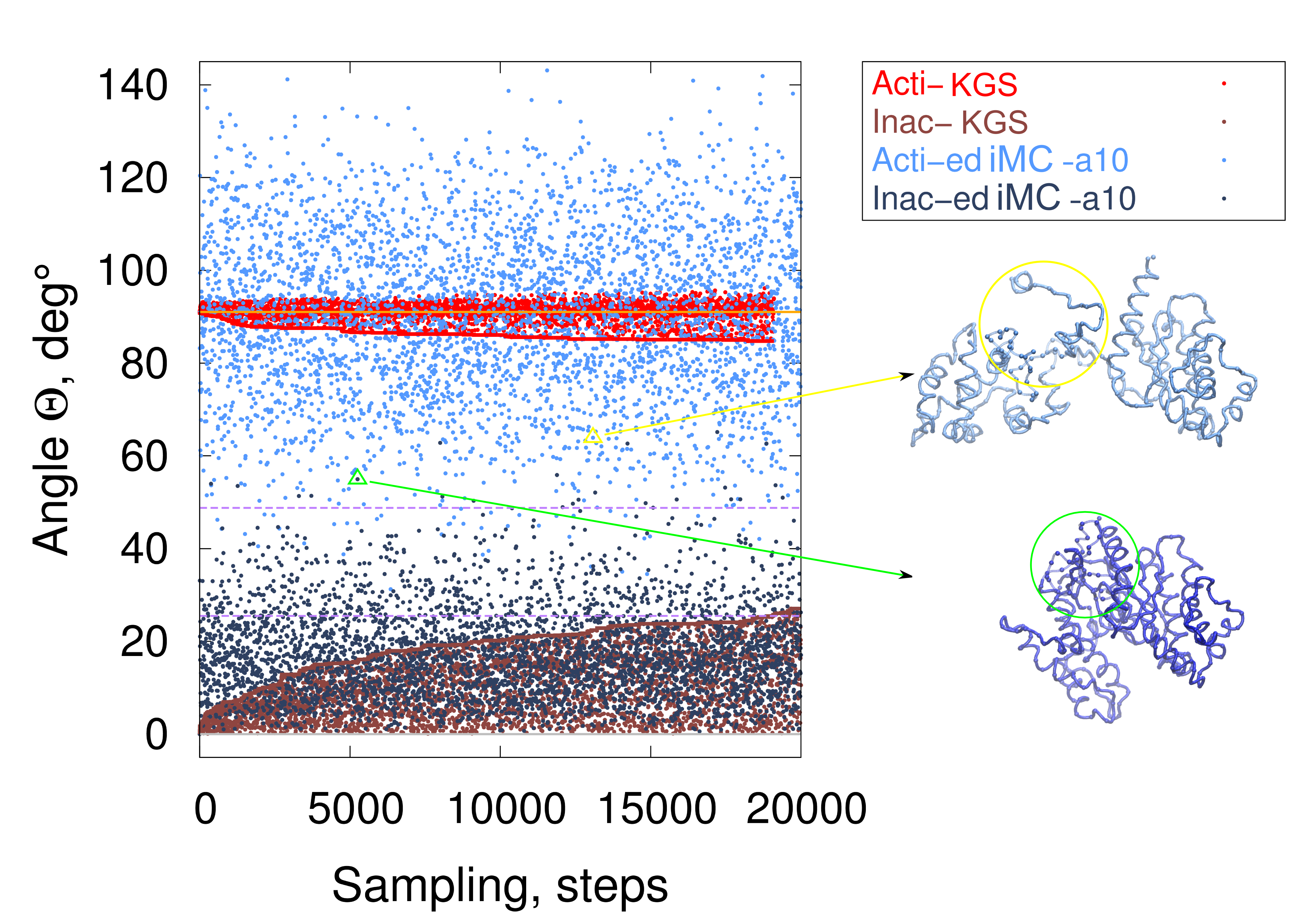

Supplement: S3 Fig — The ensembles were obtained from a coarse-grained, CA-only representation with an essential dynamics (ED) potential function. A scale factor of a = 10 was applied. The average (25.5 degrees) and maximum (48.8 degrees) opening angles for the AH domain suggested by DEER experiments are indicated by dashed horizontal lines. While the opening angle of the inactive ensemble matches that of the DEER experiment, conformations in the inactive and active ensembles are distorted. (TIFF) [file pcbi.1004361.s003.tiff]

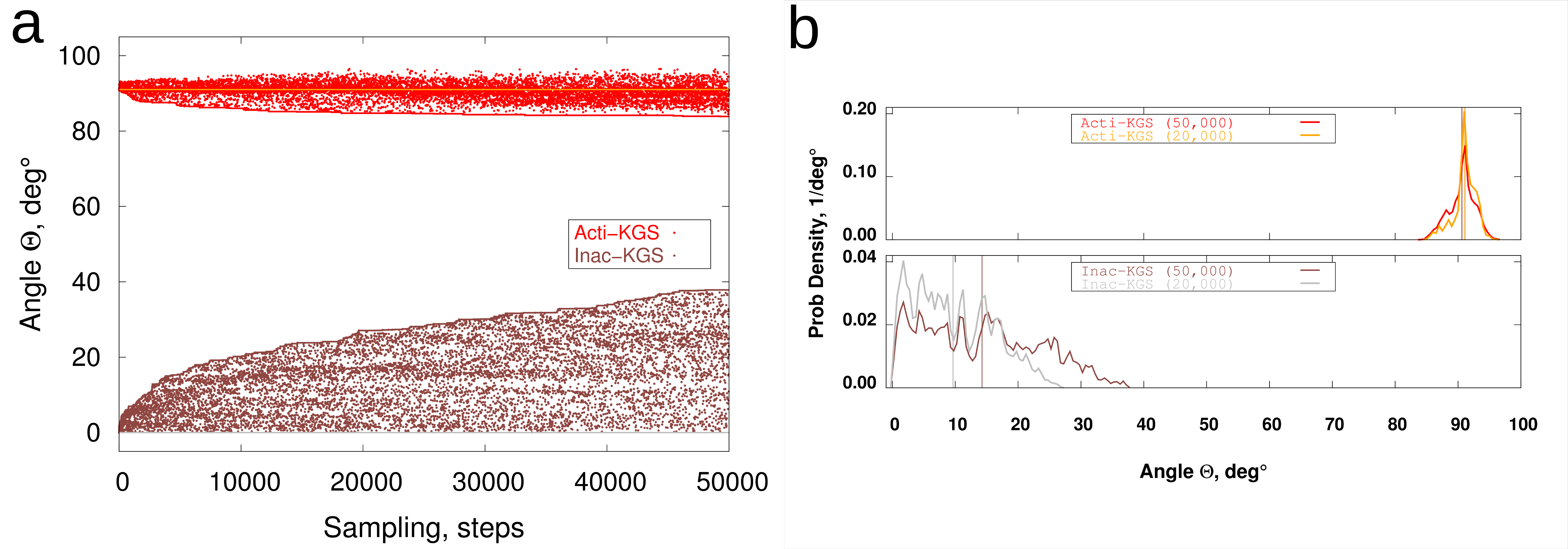

Supplement: S4 Fig — a) The change in opening angle between the AH-domain and the Ras-domain as sampling progresses for KGS. The maximum opening angle levels around 48,000 samples. b) The distributions of the opening angle for the active (top) and inactive (bottom) state from 20,000 and 50,000 samples. The active state distributions are very similar, while the inactive distributions differ in the tail. The active, 50,000 sample distribution has min = 83.9°, mean = 90.6°, and max = 96.6° opening angles. For the inactive distributions these numbers are: min = 0.0°, mean = 14.2°, max = 37.9°. (TIFF) [file pcbi.1004361.s004.tiff]

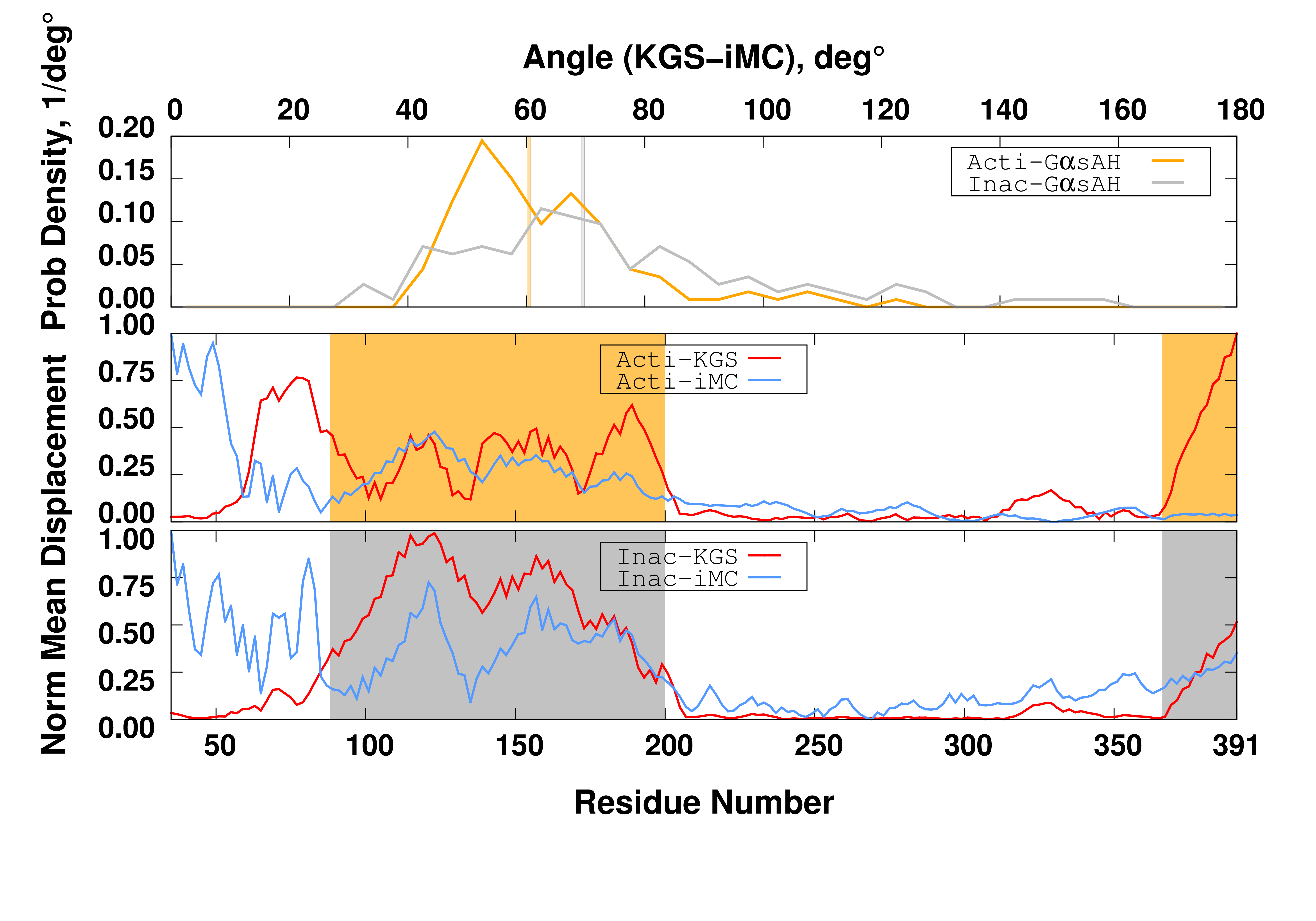

Supplement: S5 Fig — Top panel. The relative frequency of the angles between KGS and iMC average displacements for each Cα of the AH-domain in the inactive (grey) and active states (orange/yellow) is virtually unchanged in the larger KGS ensemble. Bottom two panels. The normalized magnitude of the mean Cα displacement vectors of the KGS (50,000 samples) and iMC ensembles. The longer trajectories support stronger coupling for helix α 5, but otherwise are similar to those obtained from 20,000 samples. (TIFF) [file pcbi.1004361.s005.tiff]

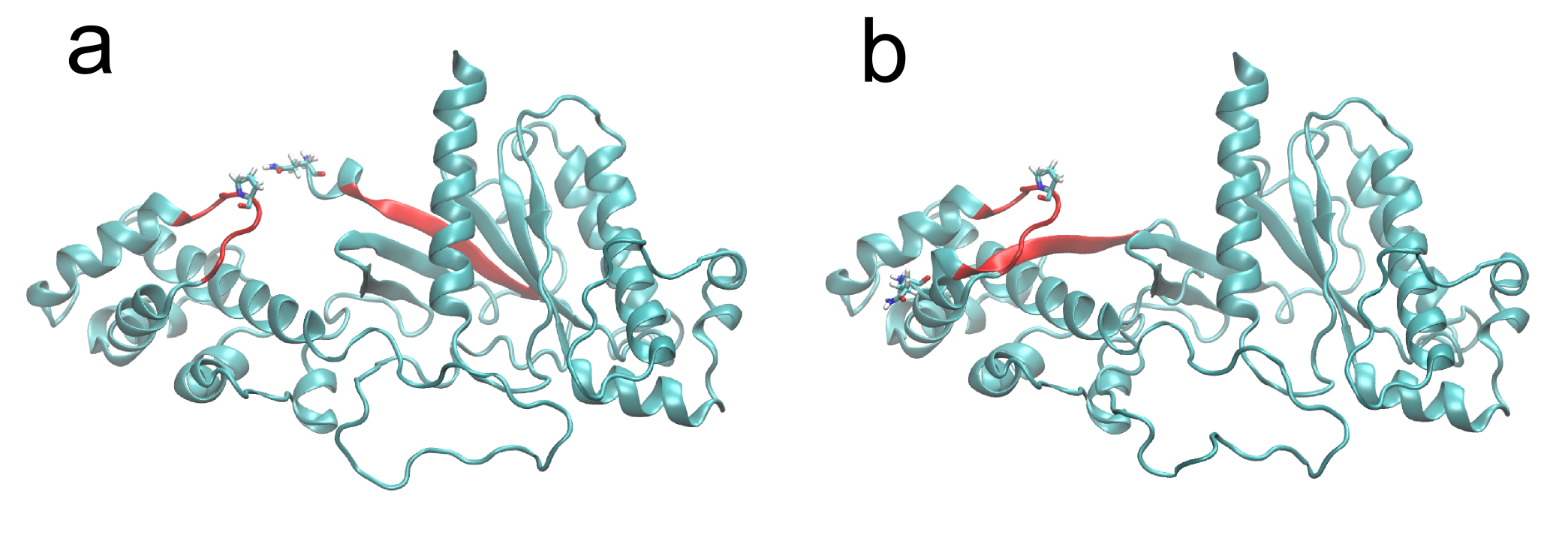

Supplement: S6 Fig — a) The proximity of the BC loop (red, with one residue in stick representation) in the AH domain and N-terminal helix α 1 (with one residue in stick representation) results in ENM restraints for the active state crystal structure. b) A snapshot of the iMC conformational ensemble. The β 1 strand (red) is coupled to the AH domain, resulting in large motion amplitudes that deform the β-sheet. (TIFF) [file pcbi.1004361.s006.tiff]
